# Decoding the Metabolic Signatures of Neurodegeneration Diseases: Advances in Mass Spectrometry-Based Metabolomics

**DOI:** 10.3390/metabo16030206

**Published:** 2026-03-20

**Authors:** Md Abdul Hakim, Li Li

**Affiliations:** 1 Clinical Pharmacology Experimental Therapeutics Center, Dallas, TX 75235, USA; 2 Department of Pharmacy Practice, Jerry H. Hodge School of Pharmacy, Texas Tech University Health Sciences Center, Dallas, TX 75235, USA

**Keywords:** neurodegenerative diseases, metabolomics, mass spectrometry, biomarker discovery, metabolomic pathways

## Abstract

The dysregulation of multiple metabolic pathways is a potential contributor to the development of neurodegenerative diseases. Understanding early-stage metabolic alterations is crucial for identifying targets associated with disease development and progression. Recent advances in mass spectrometry-based metabolomics now allow investigators to conduct a comprehensive analysis of small-molecule metabolites in complex biological systems, providing valuable insights regarding the biochemical mechanisms underlying neurodegeneration. This review presents the latest advances in mass spectrometry-based metabolomic approaches and their applications in studying neurodegenerative diseases. We discuss methodology improvements in metabolomics, including sample preparation, chromatography separations, ionization, and fragmentation. These improvements enable broader detection and more accurate identification of metabolites. We also review developments in bioinformatics tools for large-scale data processing, structural annotation, and pathway analysis. Furthermore, the signature metabolites associated with major neurodegenerative diseases and the key metabolic pathways involved are summarized. Finally, we address current analytical and biological challenges in mass spectrometry-based metabolomics while exploring its future directions in translational research.

## 1. Introduction

### 1.1. Overview of Neurodegenerative Diseases

Neurodegenerative diseases are characterized by the gradual loss of neuronal cell populations in the central and peripheral nervous systems [1,2]. This degeneration hampers communication pathways within the nervous system, resulting in impairments in memory, cognitive function, behavior, sensory, and motor abilities. The specific major causes of neurodegenerative diseases remain unknown but likely involve a complex interplay of genetic, environmental, and lifestyle factors, with aging as the primary risk factor [3,4]. Many neurodegenerative disorders exhibit early metabolomic dysregulation; for example, Alzheimer’s disease (AD) has been referred to as “type III diabetes” due to its strong association with impaired glucose metabolism in the brain [5,6]. Together, alterations in metabolic pathways and disruption of protein homeostasis ultimately lead to irreversible aggregation of key pathological proteins, such as amyloid-β and tau in AD, α-synuclein in Parkinson’s disease (PD), and transactive response DNA-binding protein of 43 kDa (TDP-43) in amyotrophic lateral sclerosis (ALS) and frontotemporal dementia (FTD) [7,8,9,10]. The burden of each of these diseases will likely increase due to the aging population worldwide [11]. For example, AD is the most common neurodegenerative disease, affecting approximately 7 million people in the US, and is projected to double by 2060 [12,13,14,15]. This highlights the crucial need for a more comprehensive understanding of the metabolic processes underlying neurodegeneration.

### 1.2. Importance of Metabolomics Study

**Figure 1:**
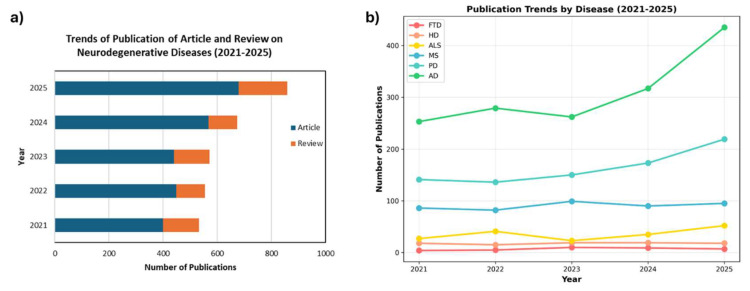
(**a**) Trends of published research articles and metabolomics review papers on neurodegenerative diseases from 2021 to 2025. (**b**) Trends of publications on different neurodegenerative diseases (AD—Alzheimer’s disease, PD—Parkinson’s disease, MS—multiple sclerosis, ALS—amyotrophic lateral sclerosis, HD—Huntington’s disease, FTD—frontotemporal dementia). Data was gathered through a literature search in the Web of Science Core Collection (WoSCC) database.

Metabolomics is the study of small-molecule metabolites (typically referring to molecular weight less than 1500 Da), which are involved in various cellular and physiological processes, such as the formation of the cell membrane, regulation of signal transduction, activation of receptors, and energy production [16,17]. Metabolomics provides an understanding of the current physiological state or alterations of metabolites in a cell, tissue, or organism. Therefore, metabolomics has become a powerful tool in the discovery of disease biomarkers, deciphering complex metabolic pathway alterations implicated by diseases, and enhancing the understanding of disease mechanisms [18,19]. The yearly trend in publications shows how research in neurometabolomics has progressed over the past five years (Figure 1). From 2021 to 2025, publications on metabolomics in neurodegenerative diseases increased substantially, rising from 532 to 858 (Figure 1a), representing an overall growth of approximately 61%. This expansion was largely driven by research articles, which grew from 399 to 679 (≈70% increase), indicating that original investigations account for most of the field’s recent development. Among individual conditions, Alzheimer’s disease (AD) received the greatest attention, with publications increasing from 253 in 2021 to 435 in 2025 (Figure 1b), mirroring the broader upward trend.

### 1.3. Advantages of Mass Spectrometry (MS)-Based Metabolomics

MS has been extensively used for analyzing metabolites because of its high resolving power and sensitivity [20]. MS can identify hundreds of thousands of molecules simultaneously by measuring their mass-to-charge ratio (*m*/*z*), with sensitivity down to the picomole and possibly sub-picomole. Small metabolites often display substantial structural diversity and distinct functional groups, yet many have very similar *m*/*z* values or, more challenging, share the same molecular formula. Chromatographic techniques such as High-Performance Liquid Chromatography (HPLC), Gas Chromatography (GC), and Capillary Electrophoresis (CE) help resolve this issue by providing distinct retention-time parameters that enable confident identification and accurate quantification. Although high-resolution MS, such as Orbitrap, Fourier Transform Ion Cyclotron Resonance (FT-ICR), and Time-of-Flight (TOF), is successful in discovering unknown molecules across a broad *m*/*z* range with mass accuracy under 1 ppm, lower-resolution triple-quadrupole mass spectrometers are better options for targeted quantitative analysis due to their high sensitivity and lower cost. Various fragmentation techniques (e.g., collision-induced dissociation, electron transfer dissociation, ultraviolet photodissociation) equipped with MS further expand its capability for structural elucidation and precise characterization of metabolites.

### 1.4. Scope and Objectives of This Review

This review summarizes current metabolomics findings related to major neurodegenerative diseases and outlines methodological considerations relevant to experimental design, including sample preparation strategies and instrument selection. Attention is given to reported metabolite alterations, candidate biomarkers, and disrupted metabolic pathways associated with disease conditions. In addition, bioinformatics tools for data processing and interpretation are discussed.

## 2. Dysregulation of Key Metabolic Pathways/Functions Involved in Neurodegenerative Disorders

### 2.1. Dysregulation in Lipid Metabolism

Lipid dysregulation (abnormal lipid metabolism, transport, or composition) is a major risk factor for neurodegenerative diseases because the brain is extremely lipid-rich and depends on tightly regulated lipid balance for structure, signaling, and energy [21,22,23]. Altered lipid metabolism has been linked to cognitive decline and the progression of neurodegenerative diseases and has been documented in many Alzheimer’s disease mouse models and postmortem human brain tissues [24,25,26].

Accumulation of arachidonic acid-containing bis(monoacylglycerol)phosphate in microglia, the brain’s immune cells, was found to be involved in the development of AD [27]. Alterations in glycerophospholipid biosynthesis and sphingolipid metabolism have been reported in human brain tissues from individuals with Alzheimer’s disease [28]. In mouse models exposed to neurotoxicants (e.g., low-dose radiation and malathion), the progression of neurodegeneration was associated with reduced biosynthesis of phosphatidylcholine and other phospholipids, as well as decreased metabolism of sphingolipids, α-linolenic acid, and linoleic acid in the hippocampus [29]. Dysregulated metabolism of hexosylceramide and phosphatidylcholine has been observed in both Alzheimer’s and Parkinson’s disease compared with healthy controls [30]. Alterations in lipid biosynthesis were also reported in PD [31]. Analysis of various brain regions in Alzheimer’s disease mice identified significant disruptions in sphingolipid, glycerolipid, and glycerophospholipid metabolism, specifically in the thalamus [32]. Furthermore, multifactorial enzyme 2 plays a critical role in the β-oxidation of fatty acids within peroxisomes, and its downregulation results in the accumulation of lipids and arachidonic acid, excessive mitochondrial ROS production, elevated proinflammatory cytokines, and the subsequent promotion of neuroinflammation [33]. The deficiency of the multifactorial enzyme 2 was observed in microglia of human AD postmortem brain tissue and AD mouse models [34]. Meanwhile, many other lipids, including ceramides, gangliosides, sphingolipids, and phospholipids, have been reported to be involved in mitochondrial dysfunction in patients with PD [35].

Cholesterol metabolism dysregulation is another common pathological feature of neurodegenerative disorders [36,37]. Cholesterol is essential for maintaining membrane fluidity and the proper formation of specialized lipid rafts. Although the precise changes differ among diseases, they frequently involve genetic risk factors (e.g., ApoE4) [37,38,39,40,41,42,43] and have been reported to affect mitochondrial function, inter-organelle communication, and glial cell responses [44]. Additionally, dysregulation in cholesterol metabolism was linked to the disruption of amyloid precursor protein processing, contributing to the progression of AD [45]. The deficiency of 24-dehydrocholesterol reductase, an enzyme regulating brain cholesterol levels, was reported to cause AD-like pathologies such as synaptic injury, neuroinflammation, tau pathology, and cognitive decline [46]. Significant alterations in cholesterol transportation proteins, including low-density lipoproteins VLDL5, LDL3, LDL4, and LDL5, were observed in both presymptomatic and symptomatic HD patients compared with controls [47]. Altered cholesterol biosynthesis and catabolism have been reported in plasma and brain tissue in Huntington’s disease models [48]. Dysregulation of key regulators, including LDLR and SREBPs, is associated with disease progression [49]. Additionally, reduced cholesterol 24-hydroxylase (CYP46A1), which converts excess brain cholesterol to 24-hydroxycholesterol, has been observed in patients [50].

### 2.2. Dysregulation in Glucose Metabolism

Glucose is the brain’s primary energy source, and impaired glucose uptake and utilization have been observed in various neurodegenerative conditions [51,52] including AD [53], PD [54], HD [55,56], and ALS [57]. Neurological symptoms, such as those in AD, can manifest after long-term dysregulation of metabolomic pathways [58,59]. Reduced glucose uptake driven by insulin resistance and inhibited insulin/insulin-like growth factor (IGF) signaling pathways [60] is strongly associated with patients with type 2 diabetes [61,62] or mild cognitive impairment (MCI) [63,64], as neurons are particularly vulnerable due to their high energy demands [65,66]. Conversely, excess glucose promotes mitochondrial oxidative stress and dysfunction [67,68,69]. In AD, reduced cerebral glucose metabolism occurs long before the onset of clinical symptoms, indicating that metabolic dysfunction could be a major driver of neurodegeneration and is not just a consequence [70]. This helps explain why diabetes is a significant risk factor for neurodegenerative diseases.

### 2.3. Dysregulation of Amino Acid and Neurotransmitter Metabolism, Among Others

Amino acids perform diverse and essential functions in the human brain, serving as precursors for hormones, neurotransmitters, and signaling molecules that support and maintain neuronal function [71] and directly influencing cognitive performance [72]. In addition, amino acids are also involved in energy production and nitrogen management [73,74]. Amino acid imbalances are now recognized as important contributors to the development of brain-related diseases, making them valuable markers for diagnosis and promising targets for medical treatment [75,76,77,78,79]. Dysregulation of alanine, aspartate, and glutamate metabolism was particularly observed in AD patients, whereas altered phenylalanine and lysine metabolism was observed in MCI patients [80]. Another study investigated plasma biomarkers of AD and reported alterations in the metabolism of arginine, alanine, aspartate, and glutamate [81]. Changes in the alanine, aspartate, and glutamate metabolism, as well as in glycine, serine, and threonine metabolism, were also reported in ALS [82]. Glutamine and gamma-aminobutyric acid metabolism alterations and their associated neurotransmitter imbalances have been reported in AD mice, which have been improved with riluzole, a glutamine modulator [83]. The kynurenine pathway (KP), which breaks down tryptophan, has also been associated with neurodegenerative diseases [84,85]. Alterations in the tryptophan-kynurenine pathway in AD were linked to disruptions in nicotinamide adenine dinucleotide metabolism, thereby affecting ATP production [86].

In addition, dysregulation of other pathways such as the inositol pathway, uronic acid pathway, purine metabolic pathway, nucleotide metabolism, and carbohydrate metabolism has been observed in AD [87,88] and MCI [89,90,91]. Disruptions in the central carbon metabolism were reported to be associated with PD [92,93]. The indole metabolic pathway, which plays a role in regulating oxidative stress and inflammation, was found to be involved in AD and PD [79,94,95].

### 2.4. Mitochondrial Dysfunction

In neurodegenerative diseases, oxidative stress in mitochondria can both drive and be a consequence of neurodegeneration [96]. In AD, mitochondrial dysfunction has been reported in both neurons and peripheral immune cells. Isolated immune cells from AD patients demonstrated altered oxidative stress in CD4+ cells, overexpression of glycolytic enzymes, and hyperpolarized mitochondrial membrane potential in CD8+ cells. These changes indicate cell-specific mitochondrial stress and metabolic imbalance in AD [97]. Tauopathy, characterized by the aggregation of tau protein, a hallmark of many neurodegenerative diseases, can also cause pronounced mitochondrial fragmentation, reduced oxygen consumption, and decreased membrane potential, ultimately leading to marked impairment of mitochondrial energy production in yeast models [98]. Overexpression of 17β-hydroxysteroid dehydrogenase 10 (HSD10), a mitochondrial enzyme, has been reported to disrupt the TCA cycle, reduce β-oxidation, and elevate oxidative stress [99]. The α-ketoglutarate dehydrogenase, an essential enzyme in the TCA cycle, converts α-ketoglutarate into succinyl-CoA and produces nicotinamide adenine dinucleotide, which is vital for ATP production [100]. The upregulation of α-ketoglutarate dehydrogenase, an essential enzyme in the TCA cycle, could lead to higher levels of α-ketoglutarate and increased mitochondrial lipid peroxidation, and this has been identified as one of the main factors of PD development [101]. In mitochondria, glutathione is the primary antioxidant defense and crucial for neutralizing reactive oxygen species (ROS). Notably, a reduction in the antioxidant glutathione was observed in the substantia nigra and left hippocampus of PD patients [102]. Glutathione was also significantly depleted in the hippocampus of AD patients compared to patients with MCI or healthy individuals [103,104,105]. Additionally, impaired peripheral mitochondrial function has been associated with primary AD pathology, although the underlying mechanisms remain unclear [106].

## 3. Advances in Technologies for MS-Based Metabolomics Study

### 3.1. Advances in Sample Preparation

**Figure 2:**
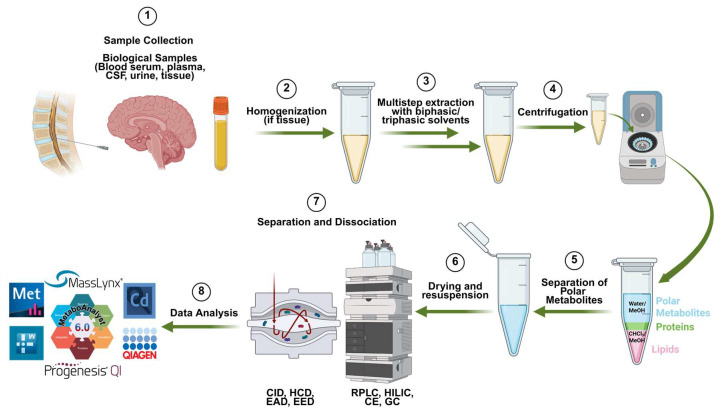
Experimental workflow of MS-based metabolomics, starting from (1) sample collection, (2–6) detailed sample preparation, (7) data acquisition, and (8) commonly used data processing software (created with BioRender).

Metabolite extraction is essential for the success of subsequent analytical analyses [107]. For polar metabolites, extraction begins with protein precipitation using ice-cold solvents such as methanol or acetonitrile, followed by centrifugation to separate a distinct layer of metabolites [108]. Lipids, mostly non-polar metabolites, can be isolated using biphasic extraction methods, such as the Folch method, or solid-phase extraction [109,110,111]. The choice of solvents, the homogenization method, and stabilization measures (like temperature control) are critical factors that can significantly improve metabolite recovery [112,113]. A detailed metabolomics workflow, including sample preparation, LC-MS analysis, and bioinformatics tools, is illustrated in Figure 2.

Chen et al. developed an efficient successive electromembrane extraction system to simultaneously extract polar and nonpolar metabolites from biological samples using a binary organic solvent mixture (2-nonanone and 2-nitrophenylpentyl ether) [114]. The method was effectively used to extract carnitine and acylcarnitines from plasma samples of an animal model with acute methcathinone poisoning. Oanes et al. optimized a salting-out-assisted liquid–liquid extraction method that used the ion-pairing reagent trifluoroacetic acid. The technique achieved high recovery rates for both polar tryptophan metabolites and non-polar bile acids from human blood serum, demonstrating its ability to handle substances with a wide range of polarities [115]. Verizian et al. investigated various extraction protocols to extract metabolites from dried blood spots of patients with phenylketonuria and found that an 80/20% acetonitrile/water solvent was the most promising [116]. Guo et al. compared monophasic and biphasic extraction techniques for extracting metabolites from cerebral tissue and reported that monophasic extraction yielded better results than biphasic extraction. In monophasic extraction, nonpolar and polar metabolites were extracted separately using isopropyl alcohol:water (77.2:23.8 *v*/*v*) and acetonitrile:methanol:water (38.1:38.1:23.8 *v*/*v*), respectively, whereas in biphasic extraction, methyl tert-butyl ether: methanol (3:1) was used, followed by re-extraction with methanol: water (1:1) [117]. Lepoittevin et al. compared three conventional solvent-based extraction methods (methanol, methanol/acetonitrile, and acetonitrile) and two hybrid solid-phase extraction methods using acetonitrile or methanol to extract plasma and serum metabolites. Among these, methanol-based extraction and solid-phase extraction provided higher metabolome coverage than other methods [118].

### 3.2. Advances in Separation Techniques

Biological samples used in metabolomics research vary widely and include blood plasma, serum, cerebrospinal fluid (CSF), urine, tissues, and cultured cell lines, among others. The complexity and abundance of metabolites can differ significantly across different sample types. Therefore, a robust and efficient separation method is crucial for distinguishing and identifying metabolites with varying polarities, volatilities, and concentrations. Liquid chromatography techniques are among the most common separation methods used in metabolomics analysis. The most common LC techniques used in metabolomics analysis include reversed-phase LC (RPLC), hydrophilic interaction LC (HILIC), and ion-exchange and mixed-mode chromatography. GC is commonly used to analyze volatile metabolites because it provides strong separation ability and highly consistent results [119]. CE, another powerful analytical technique, has been used to separate highly polar, ionic, and low-molecular-weight compounds [120]. CE separates molecules based on their charge-to-size ratio and is efficient at resolving metabolites that are often challenging to separate by LC or GC [121]. Ion Mobility (IM) is a gas-phase separation technique that separates ions based on their size, shape, and charge. IM-enhanced metabolomics has been used to perform comprehensive analyses of metabolites and distinguish their isomeric conformations [122]. Table 1 summarizes the extraction, separation, ionization, and dissociation techniques used in metabolomics, along with brief descriptions, key benefits, and drawbacks for each technique.

#### 3.2.1. Reversed-Phase LC (RPLC)

Xu et al. developed and validated two LC-MS/MS methods using reversed-phase and HILIC chromatography to quantify 235 metabolites in porcine plasma samples without derivatization. This 40 min multi-step gradient allowed high metabolite coverage and demonstrated robustness for large-scale targeted metabolomics [123]. Subramaniyan et al. optimized an RPLC-MS method to separate structurally similar oxysterols and successfully quantified eight different oxysterols simultaneously. The method was applied to investigate alterations in oxysterol levels in mice fed a high-fat diet compared with a regular diet [124]. Zhu et al. developed an untargeted metabolomics method using RPLC-MS and optimized parameters, including injection volume and reconstitution solvent, to enhance metabolome coverage. Additionally, a post-column infusion approach was employed to monitor both absolute and relative matrix effects during the analysis of plasma and fecal samples [125]. Another study tested ion chromatography-MS, RPLC-MS, and HILIC-MS methods to assess the correction of ion suppression using a stable isotope-labeled internal standard [126]. RPLC has also been used to examine the effect of formic acid pretreatment on analytical performance in untargeted metabolomics, where they found that this pretreatment significantly improves sample preparation reproducibility and signal intensity [125]. Hooshmand et al. compared the efficiency of different extraction methods for metabolite extraction from human CSF, used RPLC-MS and HILIC-MS to separate and characterize the metabolites, and identified a total of 674 unique metabolites with the optimized method [127].

#### 3.2.2. Hydrophilic Interaction LC (HILIC)

Li et al. developed a simple, rapid, and sensitive HILIC-MS/MS method for the simultaneous identification and quantification of purine metabolites. The technique enabled efficient separation and quantification of 16 structurally similar nucleosides and deoxynucleosides. The biological applicability of the method was tested using plasma and urine samples from acute kidney patients, in which an abnormality in the purine metabolism pathway was found [128]. Another study to investigate purine metabolism in cultured cells presented the development of an HILIC-MS/MS method, which enabled the simultaneous determination of canonical purine metabolites without derivatization [129]. A study comparing different extraction methods and separation techniques for milk metabolites found that methanol-based extraction yielded better recovery and that the HILIC column provided the best separation of metabolites. Out of the fifteen columns tested, the HILIC column provided better retention and separation of the milk metabolites [130].

#### 3.2.3. Ion-Exchange and Mixed-Mode Chromatography

Grubner et al. developed a comprehensive 2D-LC method combining a mixed-mode reverse-phase/ion-exchange and a HILIC for the separation of urine metabolites. This method offered broader coverage of metabolites, including polar, moderately polar, and non-polar metabolites [131,132]. Correia et al. introduced a mixed-mode liquid chromatography method combining anion exchange and hydrophobic interactions within a single stationary phase and demonstrated comprehensive separation of metabolites across a broad polarity range in a 4 min run [133]. Xing et al. used a positively charged quaternary amine polyvinyl alcohol stationary phase to design a mixed-mode chromatography method in order to separate the metabolites derived from the central carbon metabolism. The stationary phase retained 398 of 607 unique metabolites tested. The method also showed the separation of glucose from fructose and four hexose monophosphates [134].

#### 3.2.4. Gas Chromatography (GC)

Zeki et al. presented an optimized GC-MS method for untargeted metabolomics, comparing three GC gradients: short (26.7 min), standard (37.5 min), and long (60 min). While the long GC method showed improved resolution and increased metabolite coverage, the short method was suited for high-throughput metabolomics analysis [135]. Wang et al. developed a GC-MS method for qualitative and semi-quantitative metabolomics analysis. The method provided high metabolite coverage with the accuracy and robustness of targeted analysis. With this method, they were able to establish a strong relationship between the retention time of straight-chain fatty acid methyl esters and their retention time indices in the existing database [136]. Huang et al. optimized a derivatized GC-MS method comprising methanol extraction, ultrasonication, and derivatization with N-Methyl-N-(trimethylsilyl)trifluoroacetamide for the analysis of non-volatile metabolites. While analyzing the same set of samples using nuclear magnetic resonance (NMR) and GC-MS methods, 63 metabolites were identified using GC-MS, and only 24 metabolites were detected in NMR [137].

#### 3.2.5. Capillary Electrophoresis (CE)

An untargeted CE-MS method was optimized using a capillary coated with polyvinyl alcohol to analyze highly polar and negatively charged metabolites without prior derivatization. The method was successfully used to identify amino acids, amino acid derivatives, carboxylic acids, organic acids, sugars, and phosphoderivatives of sugars [138]. Zaripov et al. investigated alterations in purine and carnitine metabolism in breast cancer exosomes using a nanosheath–flow capillary electrophoresis–MS system [139]. Narduzzi et al. used both HILIC-MS and CE-MS to effectively separate polar metabolites from pig serum exposed to environmental pollutants, polychlorinated biphenyls. Results showed that combining HILIC-MS and CE-MS offered broader metabolome coverage [140].

#### 3.2.6. Ion Mobility Spectrometry (IMS)

Ion mobility spectrometry provides additional separation to distinguish challenging isomeric metabolites in the gas phase. Zhang et al. introduced a robust analytical method that combines chiral derivatization with differential ion mobility spectrometry (DIMS) to distinguish amino acid enantiomers. N-(4-nitrophenoxycarbonyl)-l-phenylalanine 2-methoxyethyl ester was used as a chiral derivatization reagent, and the derivatized amino acids were separated and detected using DIMS-MS. A total of 11 enantiomeric amino acids were baseline separated using this method [141]. Kingsley et al. employed high-resolution structures for lossless ion manipulations, an ion mobility separation technique, to achieve better separation of vitamin D metabolites and their isomers. With a high resolving power (about 200), the technique successfully identified previously unresolved conformations for several compounds, including 25-hydroxyvitamin D2 and its epimers, epi-25-hydroxyvitamin D2, and 1,25-dihydroxyvitamin D3 [142].

### 3.3. Advances in Ionization Techniques

Ionization techniques are essential to MS-based metabolomics because they significantly impact sensitivity, metabolite coverage, and quantitative accuracy [143]. Among these techniques, matrix-assisted laser desorption ionization (MALDI), desorption electrospray ionization (DESI), and electrospray ionization (ESI) are the most frequently used. MALDI has been extensively employed in MS imaging for direct visualization of metabolites within tissues [144]. As a versatile ambient ionization technique for mass spectrometry imaging, DESI has demonstrated the ability to detect a wide range of lipids and metabolites [145]. ESI, another commonly used ionization method, effectively ionizes a broad range of metabolites and provides improved sensitivity for detecting low-abundance metabolites.

Chen et al. developed a new dual-polarity MALDI matrix using 4-aminocinnoline-3-carboxamide and compared it with traditional matrices such as 2,5-dihydroxybenzoic acid (DHB) and 9-aminoacridine. MALDI imaging with the new matrix showed improved performance when investigating the transgenic AD mouse brain. The study identified 93 regionally altered metabolites in AD mice compared with healthy controls, providing insights into AD pathogenesis through changes in metabolic pathways [146]. Zhang et al. optimized a MALDI MS imaging method for direct and rapid visualization of cholesterol distribution in AD and cancer tissue. They optimized specific parameters, such as slice thickness, matrix selection, matrix deposition method, and deposition thickness, as well as instrument settings such as laser spot size, laser intensity, and mass-to-charge range. The optimized method demonstrated improved ionization efficiency for cholesterol and also revealed significant upregulation of cholesterol in the AD mouse cerebellum compared to the wild-type mouse [147].

Lv et al. developed segmented temperature-controlled desorption electrospray ionization (STC-DESI), a refined mass spectrometry imaging platform that achieves a cellular-level spatial resolution of 20 μm by accurately controlling desorption and ionization temperatures. By applying this technique to transgenic AD mouse models, they observed significant molecular differences around individual Aβ plaques, including increased sulfatides and decreased small-molecule metabolites like carnosine. This highly sensitive, label-free imaging method proved to be capable of revealing localized pathological changes in the early stages of Alzheimer’s disease [148]. Rahman et al. developed a new ionization enhancement method for desorption electrospray ionization-mass spectrometry imaging by using low-temperature plasma (LTP) pretreatment to address the typically poor ionization of brain cholesterol. By exposing brain sections to LTP for one minute before analysis, the study achieved a twofold increase in cholesterol signal intensity and effectively distinguished it from isomers using multiple reaction monitoring (MRM). This method successfully mapped high cholesterol levels within white matter fiber tracts, such as the corpus callosum and anterior commissure, while also enabling the detection of previously unobservable analytes [149].

Xu et al. developed a hybrid ionization source by combining nanoelectrospray ionization and atmospheric pressure chemical ionization (nanoESI-APCI), which demonstrated roughly 10 times the sensitivity of nanoESI alone. The optimized method, when applied to single-cell metabolomics, detected 254 metabolites compared to only 172 identified with nanoESI. The technique was also successfully used to study cancer cell metabolism and cellular responses to glucose starvation [150]. Girel et al. introduced a microflow liquid chromatographic system paired with a microfabricated multinozzle electrospray emitter to boost ionization efficiency and sensitivity. This innovative setup, which combined five nozzles operating at 600 nL/min each, showed significantly higher sensitivity than traditional ESI. A 19 min analysis of deuterated lipid standards using this setup yielded a roughly 16-fold median increase in signal compared to conventional analytical flow ESI. When applied to a 3D clear cell renal cell carcinoma model exposed to a multidrug combination therapy, the new method identified 1270 lipids, while only 752 were detected with analytical flow ESI [151]. Nguyen et al. developed a lithium-doped nanospray desorption electrospray ionization method that significantly improved the ionization efficiency of metabolites and lipids that lack basic groups and are difficult to detect in positive ion mode. Additionally, adding lithium to the ESI solvent boosted signal intensities by 10–1000 times for metabolites, fatty acids, phospholipids, and neutral lipids [152].

### 3.4. Advances in Fragmentation Techniques

Advanced fragmentation techniques in MS are crucial for elucidating metabolite structures and distinguishing isomeric species, offering much more detailed molecular insights than basic mass measurements alone. Although traditional methods such as collision-induced dissociation (CID) and higher-energy collisional dissociation (HCD) are widely used, they often have limitations when analyzing structurally complex metabolites [153]. To address these challenges, electron-based dissociation techniques—electron-capture dissociation (ECD), electron-transfer dissociation (ETD), electronic excitation dissociation (EED), and electron-activated dissociation (EAD)—have been introduced as highly effective, complementary techniques [154]. EAD, for example, produces unique fragment ions via alternative, orthogonal pathways, providing improved structural clarity and surpassing conventional CID and HCD in many complex analytical cases [155].

Tang et al. developed an electronic excitation dissociation (EED) method to identify isomeric glucuronide structures at the MS^2^ level. While the traditional CID method could not provide information on the glucuronidation linkage, the EED method distinguished the isomers by unique MS^2^ fragments, eliminating the need for additional derivatization. The method was effectively used to characterize acyl-, N-, and O-glucuronide isomers [156]. Gao et al. demonstrated the structural characterization of acylcarnitines, which are metabolites of fatty acids, using electron-activated dissociation techniques. Using this dissociation technique, they localized methylation sites, hydroxyl groups, and acyl side chains in acylcarnitines and revealed alterations in isomeric acylcarnitines in type 2 diabetic mouse models [157]. Another study also highlighted the advantages of EAD over CID for the structural elucidation of conjugated drug metabolites. Using rat liver microsomal incubations, conjugation products such as glucuronides and glutathione adducts were generated and analyzed by high-resolution MS/MS with both EAD and CID. Compared to CID, EAD produced more unique fragments for most conjugates by cleaving the relatively stable bond on the parent drug while preserving the weaker conjugation bond [158]. EAD was also used to identify and localize the glucuronidation and oxidative metabolism sites of drugs [159].

## 4. Data Acquisition and Analysis

### 4.1. Targeted Metabolomics

Quantitative analysis of metabolites is essential for understanding biological processes and disease mechanisms and for discovering potential disease biomarkers. Measuring the absolute concentrations of metabolites is generally considered the most reliable and consistent method for quantitative metabolomics because it enables direct comparison of results across studies [17]. In contrast, relative quantification relies on signal intensities, which can vary significantly between analytical batches, even when the same methods are used, making it hard to combine data from multiple experiments. Although absolute quantification addresses this issue, accurately measuring endogenous metabolite levels in complex biological samples remains challenging [160]. Both biological and analytical variability must be carefully corrected to obtain accurate quantitation. Usually, biological variability is managed through normalization processes performed before data collection, while variations caused by the analytical workflow are corrected through post-acquisition normalization before further data analysis [161,162].

Stable isotope-labeled internal standards (SIL-IS) with properties similar to those of the analytes of interest are often used for accurate and robust quantitation of metabolites. SIL-IS can also be spiked directly into the samples to determine the concentrations of specific analytes. Although SIL-IS is effective for correcting the matrix effect and enabling accurate absolute quantitation, it is expensive, and its availability is limited. Zhu et al. developed a method using HILIC-MS in conjunction with post-column infusion of standards to perform the absolute quantification of polar metabolites without the need for SIL-IS. The post-column infusion of standards was performed by continuously infusing standards after chromatographic separation through a T-junction before MS detection. The method produced results similar to those from the analysis with SIL-IS and was even better for analytes that did not have a matching SIL standard [163]. Wang et al. developed a quantitative metabolomics method using isotopically ^13^C-labeled yeast extract as an internal standard to investigate changes in spatial metabolomic profiles in the brain and kidney tissues of mice experiencing stroke. The method showed significant differences in some key metabolites, such as lysine, glutamine, uridine diphosphate, N-acetylglucosamine, and linoleate, whereas traditional normalization without internal standards was unable to detect these differences [164]. Fu et al. developed a targeted metabolomics method to measure metabolites associated with pathways such as TCA, glycolysis, and oxidative phosphorylation. Using this method, they were able to simultaneously quantify 31 endogenous metabolites. The method was applied to study metabolic dysfunction associated with fatty liver, and seven metabolites—citrate, α-ketoglutarate, lactate, fumarate, succinate, malate, and glucose-6-phosphate—showed differential expression, suggesting they could serve as potential biomarkers for the disease [165].

### 4.2. Untargeted Metabolomics

**Figure 3:**
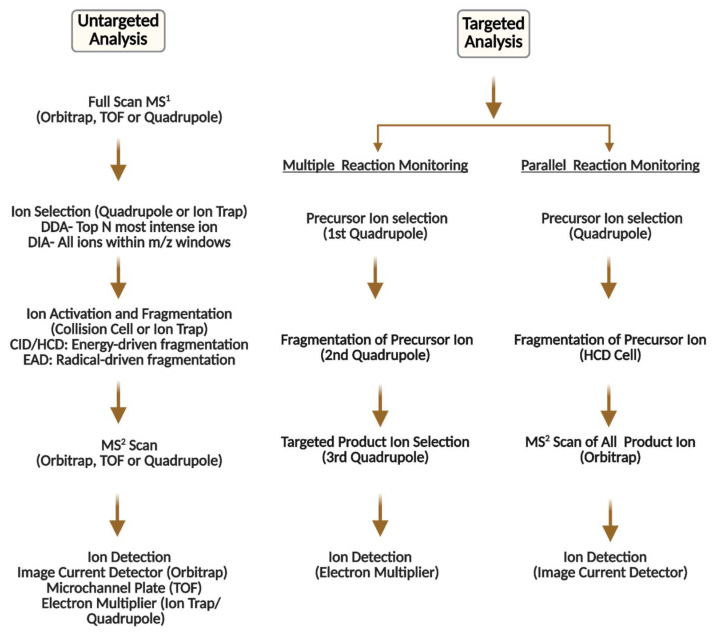
Typical MS-based metabolomics workflow of untargeted and targeted analysis.

The goal of untargeted metabolomics is to perform a comprehensive analysis of all small-molecule metabolites in a biological sample, providing a broad view of metabolic activities and changes without focusing on specific targets. Although this approach is highly effective for discovering new biomarkers and comparing groups, achieving accurate measurements remains a significant challenge [19]. Unlike targeted methods, which use specific standards to determine exact concentrations, untargeted studies typically produce relative or semi-quantitative data that can be affected by various factors during the experiment [166]. Untargeted metabolomics approaches have been widely used to discover new biomarkers for neurodegenerative diseases. Figure 3 illustrates the step-by-step process of MS-based untargeted and targeted metabolomics.

Oka et al. used untargeted metabolomics to identify significantly altered metabolites, and 93 metabolites were identified, including ganglioside GM3 and lysophosphatidylcholine, which were highly upregulated in AD [167]. Chang et al. investigated the hair metabolome of 5xFAD mice and identified 45 metabolites that were altered in AD compared to non-demented controls. Among these metabolites, L-valine and arachidonic acid were most significantly expressed and can be considered potential biomarkers of AD [168]. Ambeskovic et al. employed an untargeted metabolomics approach to find region-specific markers of AD in the human post-mortem brain. Across the 8 regions investigated, the changes in Brodmann area 9 were most pronounced. Several neurotransmitters, including phenylalanine, phosphorylcholine, N-acetylaspartate, and gamma-aminobutyric acid, were found to be significantly different in AD compared to healthy subjects [169]. Liu et al. investigated metabolic changes in plasma and fecal samples from PD patients using an untargeted metabolomics method and identified ten significant metabolites that were highly upregulated in plasma from PD patients compared to healthy controls, among which 3,4-dihydroxyphenylglycol O-sulfate and propyl gallate had been previously reported in PD cases [170]. Chen et al. reported 144 dysregulated plasma metabolites in PD patients, with sodium deoxycholate, S-adenosylmethionine, L-tyrosine, 3-methyl-L-tyrosine, 4,5-dihydroorotic acid, 6-octadecenoic acid, and allantoin showing the highest diagnostic ability to distinguish PD from controls [93]. Wang et al. discovered several plasma biomarkers of PD, including phosphatidylcholine, eicosatrienoic acid, pentalenic acid, and aspartic acid, using comprehensive untargeted metabolomics and lipidomics approaches [171].

### 4.3. Pseudotargeted Metabolomics

Pseudotargeted metabolomics is an integrated analytical method that combines the broad coverage of untargeted methods with the precise quantification of targeted methods, allowing for simultaneous high-throughput detection and accurate measurement of metabolites in complex biological samples [172]. The process starts by using high-resolution mass spectrometry to generate a comprehensive list of ion pairs (precursor and product ions) from a biological sample. These pairs are then monitored using a triple quadrupole mass spectrometer in MRM mode [173]. This transition enables researchers to achieve greater sensitivity, a broader dynamic range, and improved quantitative repeatability compared with traditional untargeted profiling, making it especially suitable for large-scale clinical cohorts and biomarker discovery [174].

Li et al. developed a pseudotargeted metabolomics method using GC-MS/MS. The method incorporated a sample-specific MS library and a pseudotargeted MRM list with 227 metabolites to detect new metabolites in new samples. As the MRM list was dynamically updated to add newly discovered compounds, the method was effectively used to identify and quantify more than 500 metabolites. The result also demonstrated that this method significantly improved metabolite coverage, identifying 33–40% of metabolites exclusively through the dynamic MRM target list [175]. Xiao et al. developed a novel pseudotargeted metabolomics approach to identify and quantify free fatty acid isomers in beagles. The method used a two-step derivatization: epoxidation to locate the double bonds and amidation to improve MS sensitivity, followed by an MRM experiment. This method successfully quantified 30 distinct free fatty acid isomers in beagle plasma samples, most of which were difficult to detect using standard protocols [176]. Huang et al. introduced a comprehensive pseudotargeted metabolomics workflow in which two-phase extraction (aqueous and organic) was performed using LC-MS/MS to extract a broad range of metabolites. Combining both extracts into a single injection, 486 metabolites were identified using this method. This method increased the metabolite coverage by over 20% compared to the traditional methanol-based protein precipitation method. This approach provided a highly sensitive, time-efficient alternative that offered extensive metabolite coverage for biomarker discovery with the accuracy of targeted analysis [177].

### 4.4. Bioinformatics and Data Analysis in Metabolomics

Effective bioinformatics tools and data processing workflows are crucial to metabolomics because they convert raw MS outputs into biologically meaningful insights. The workflow begins with data preprocessing, which includes peak detection, alignment, normalization, and deconvolution. These steps assist in handling the high complexity and variability of metabolomics datasets [178]. Modern alignment tools have the capability to correct retention time shifts and batch-to-batch variation, improving reproducibility in large studies [179]. Normalization is equally important for minimizing noise arising from instrument drift, differences in sample handling, or biological heterogeneity. Accurate metabolite annotation remains one of the most challenging aspects of metabolomics due to incomplete spectral libraries and the presence of many unknown features. Combining information from multiple databases, such as HMDB, KEGG, PathBank, MetaboAnalyst, MassBank, Metlin, Lipid Maps, and ChEBI, along with in silico prediction tools and machine-learning models helps to expand annotation coverage [180,181,182,183,184,185,186]. Machine learning and Artificial Intelligence are also increasingly crucial for identifying metabolic patterns associated with neurological diseases [187,188]. Pathway and network analysis tools offer a systems-level understanding of neurological dysfunction by placing metabolite changes within a biochemical and molecular context.

Some commonly used software for untargeted metabolomics, developed by different vendors, include Compound Discoverer 3.5 (Thermo Fisher Scientific, Waltham, MA, USA), MassLynx 4.2 (Agilent Technologies, Santa Clara, CA, USA), Markerview 1.3.1 (AB SCIEX, Framingham, MA, USA), MetaboScape 2026 (Bruker, Billerica, MA, USA), and ProGenesisQI 2.0 (Waters, Milford, MA, USA). A list of recently developed or upgraded web-based metabolomics data processing and analysis software is included in Table 2.

### 4.5. MS-Based Metabolomics in Studying Neurodegenerative Disease

Metabolomics has become a powerful tool for detecting biochemical alterations that may enable earlier diagnosis and better clinical management of neurodegenerative diseases. Current research has revealed several factors that contribute to disease development, including impaired mitochondrial function, increased oxidative stress, abnormal regulation of cell death, and disruptions in carbohydrate and lipid metabolism [203]. Common metabolic markers include branched-chain amino acids, various lipid species, acylcarnitines, and metabolites involved in different pathways. Table 3 summarizes recent studies on various neurodegenerative diseases, affected metabolic pathways, altered metabolites, separation techniques, types of MS used to detect the metabolites, and key research findings.

Alterations in various amino acids were observed in a study of the serum from patients with AD and vascular dementia [104]. Among the 29 amino acids investigated, significant upregulation of creatine and spermidine and downregulation of tyrosine, histidine, creatinine, and ornithine were found in AD compared to healthy controls. Tyrosine and ornithine remained significantly lower in AD than in vascular dementia. Dysregulation of tyrosine has also been reported in other studies on AD, PD, and MS [79,93,204,205,206]. Chu et al. investigated the dysregulated amino acids and carnitine metabolites in patients with MCI and dementia to find potential biomarkers for the early detection and diagnosis of AD [207]. A total of 36 amino acids and carnitine metabolites were found to be dysregulated, including aspartic acid and serine, which were significantly elevated in MCI and dementia. Another study on AD reported alterations in 18 amino acids, with L-glutamine and L-asparagine levels being more pronounced and significantly increased in AD [208]. Glycine, glutamic acid, and beta-alanine were found to be dysregulated in patients with ALS [82]. Cysteine-S-sulfate, N-acetyl aspartic acid, and 3-N-acetyl tryptophan were found to be altered in PD [209]. Among the metabolites derived from the kynurenine pathway (KP), neuroprotective compounds such as kynurenine, tryptophan, and anthranilic acid were reduced, while neurotoxic metabolites like 3-hydroxyanthranilic acid were significantly increased in AD [210]. KP metabolites have also been altered in ALS, where anthranilic acid was downregulated, and a lower kynurenine-to-tryptophan ratio was observed [211]. A study on HD examined its connection to dysregulation of KP metabolites, but no significant differences were found between HD and healthy controls [212]. Indole derivatives, including indole-3-acetic acid and 5-hydroxyindoleacetaldehyde, were significantly increased in PD, and indole was notably upregulated in AD [79,94,95].

Dysregulation of lipids is another common feature of neurodegenerative diseases. Dysregulated glycerophospholipid and sphingolipid isomers (GDla and GD1b) have been reported in several AD cases [28,77,78,205,213]. Dysregulation of short-chain fatty acids, dodecanoic acid, and arachidonic acid was reported in patients with AD, PD, and ALS [105,208,214,215,216]. Increased levels of ganglioside GM3 and ceramide and decreased levels of phosphatidylethanolamine and sphingomyelin were found in plasma from patients with FTD [217]. Upregulation of propionylcarnitine, lysophosphatidylcholine, taurodeoxycholic acid, and tauroursodeoxycholic acid and downregulation of hexceramide, hexadecatrienoic acid, and phosphatidylcholine were reported in AD-dementia with insulin resistance [205].

**Table 3:** List of recent studies on neurodegenerative diseases, including affected metabolic pathways, altered metabolites, separation techniques, types of mass spectrometry used to detect the metabolites, and key research findings. Abbreviations: Alzheimer’s disease (AD), Frontotemporal dementia (FTD), Parkinson’s disease (PD), amyotrophic lateral sclerosis (ALS), Huntington’s disease (HD), and multiple sclerosis (MS).

Index (Citation)	Year of Publication	Diseases	Metabolomic Pathways/Metabolites	Separation Method	Mass Spectrometry Model	Key Findings
1. [104]	2025	AD	Glutathione metabolism, arginine metabolism	LC	QTRAP 5500 (AB SCIEX, Redwood City, CA, USA)	Upregulation of creatine and spermidine and downregulation of aminoadipic acid, tyrosine, histidine, creatinine, and ornithine
2. [105]	2025	AD	Gamma-aminobutyric acid, short-chain fatty acid	GC	GC-MS7890B-7000D (Agilent Technologies, Oregon)	Depletion of short-chain fatty acid
3. [218]	2025	AD	Acetylcholine	LC	QTOF 6546 (Agilent Technologies, Waldbronn, Germany)	Altered acetylcholinesterase enzyme activity
4. [219]	2025	AD	Urocanic acid, gluconic acid, glycerophosphocholine, citicoline	LC	Q Exactive Orbitrap (Thermo Fisher Scientific, San Jose, CA, USA)	Dysregulated cerebral lipid metabolism, energy metabolism, and oxidative stress
5. [207]	2025	AD	Asp, Ser, carnitine metabolites (C5:1, C12, C14DC, C5DC/C16, and C8/C10)	LC	QTRAP 4500 (AB SCIEX, Redwood City, CA, USA)	Validated seven dysregulated metabolites as a biomarker for early detection of AD
6. [28]	2025	AD	Glycerophospholipid and sphingolipid metabolism	LC	Q Exactive Orbitrap (Thermo Fisher Scientific, San Jose, CA, USA)	Dysregulation of ganglioside isomers, GD1a and GD1b
7. [94]	2025	AD	Retinol metabolism	LC	Q Exactive HF Orbitrap (Thermo Fisher Scientific, San Jose, CA, USA)	Significant alteration of theophylline, vanillylmandelic acid, adenosine, 1,7-dimethyluric acid, cystathionine, and indole
8. [204]	2025	AD	The alanine, aspartate, and glutamate pathway	LC	SYNAPT G2, QTOF (Waters Inc., Manchester, UK)	Significant upregulation of phenylalanine, tryptophan, and tyrosine
9. [205]	2025	AD	Glycerophospholipid metabolism, glucose metabolism	LC, GC	Triple Quadrupole 6490 (Agilent Technologies, Santa Clara, CA, USA)	Upregulation of propionylcarnitine, lysophosphatidylcholine, taurodeoxycholic acid, and tauroursodeoxycholic acid and downregulation of hexceramide, hexadecatrienoic acid, phosphotidyl choline, and vanillylmandelic acid
10. [214]	2025	AD	Fatty acid metabolism, energy metabolism	GC	GC/MS 5977B (Agilent Technologies, Santa Clara, CA, USA)	Significant downregulation of dodecanoic acid
11. [87]	2025	AD	Glucose-6-phosphate metabolic pathway, glutathione metabolic pathway	LC	Q Exactive hybrid quadrupole Orbitrap (Thermo Fisher Scientific, San Jose, CA, USA)	Interactions of acetylcholine with choline O-acetyl transferase and choline transporters
12. [210]	2024	AD	Kynurenine pathway	LC	Shimadzu Triple Quadrupole 8050 (Shimadzu, Japan)	Dysregulation of 3-hydroxyanthranilic acid, quinolinic acid
13. [213]	2024	AD	Glycerophospholipid metabolism	LC	6560 IM-QTOF (Agilent Technologies, Santa Clara, CA, USA)	Alteration of glycerophospholipid sn-isomers in different regions of the AD brain
14. [77]	2024	AD	glycerophospholipids and sphingolipids metabolism, amino acid metabolism	LC	OrbiSIMS (National Physical Laboratory, Teddington, UK)	Dysfunction in amino acid and tRNA aminoacylation metabolic processes
15. [88]	2024	AD	Inositol pathway, uronic acid pathway, TCA	GC	ShimadzuQP2020single quadrupole (Shimadzu, Japan)	Impaired phosphorylation of glucose
16. [78]	2023	AD	Fatty acyls, glycerolipids, glycerophospholipids	GC	Agilent Accurate-Mass Q-TOF 6520 (Agilent Technologies, Santa Clara, CA, USA)	Altered lipid and amino acid metabolism and an imbalance of metabolites associated with energy metabolism
17. [208]	2023	AD	Malic acid, monoacylglyceride, L-asparagine, L-glutamine, D-galactose, D-arabitol, glycerol, linolelaidic acid, glycolic acid	GC	Agilent 5977A MSD (Agilent Technologies, Santa Clara, CA, USA)	Carbohydrate metabolism deficiency and dysregulation of amino acids, fatty acids, and lipid metabolism
18. [86]	2021	AD	Tryptophan- kynurenine pathway	LC	Agilent 6495 Triple Quadrupole (Agilent Technologies, Santa Clara, CA, USA)	Alterations in NAD+ metabolism
19. [217]	2025	FTD	Gangliosides, ceramide, polyunsaturated triacylglycerol	LC	Q Exactive Orbitrap (Thermo Fisher Scientific, San Jose, CA, USA)	Alterations of sphingolipids
20. [92]	2025	PD	Ergocalciferol, glutaric acid, ephedrine, guanine	LC	Q Exactive Orbitrap (Thermo Fisher Scientific, San Jose, CA, USA)	Altered metabolic profile and purine metabolic pathway
21. [220]	2025	PD	S-(1,2-dichlorovinyl)-glutathione, S-(1,2-dichlorovinyl)-L-cysteine, N-acetyl-S-(1,2-dichlorovinyl)- L-cysteine	LC	Q-Exactive Focus Hybrid Quadrupole-Orbitrap (Thermo Fisher Scientific, San Jose, CA, USA)	Elevated levels of trichloroethylene glutathione conjugation metabolites
22. [93]	2025	PD	Sodium deoxycholate, S-adenosylmethionine, L-tyrosine, 3-methyl-L-tyrosine, 4,5-dihydroorotic acid, (6Z)-octadecenoic acid, allantoin	LC	Orbitrap Exploris 120 (Thermo Fisher Scientific, San Jose, CA, USA)	Disruption of central carbon metabolism and inactivation of the peroxisome proliferator-activated receptor signaling pathway
23. [209]	2024	PD	cysteine-S-sulfate, 1-methylxanthin, vanillic acid, N-acetyl aspartic acid, 3-N-acetyl tryptophan, 5-methoxytryptophol	LC, GC	Sciex TripleTOF 6600, Leco Pegasus HT TOF (AB SCIEX, Redwood City, CA; Leco Pegasus, St. Joseph, MI, USA)	Dysregulated lipid metabolism and alteration of several key metabolites leading to neuroinflammation and neuronal damage
24. [221]	2024	PD	2-Methoxyestradiol, hydrogen peroxide	LC	Shimadzu Triple Quadrupole 8050 (Shimadzu, Japan).	Elevated level of 2-methoxyestradiol associated with neuronal damage
25. [79]	2024	PD	Amino acid metabolism, caffeine metabolism, purine metabolism	LC	Q Exactive Orbitrap (Thermo Fisher Scientific, San Jose, CA, USA)	Dysregulation of 12 metabolites, including dehydroepiandrosterone sulfate, pipecolic acid, N-acetyl leucine, 2-aminoadipic acid, L-tyrosine, uric acid, and 5-hydroxyindoleacetaldehyde
26. [95]	2023	PD	Indole metabolic pathways	LC	Triple quadrupole API 3200 (Applied Biosystems Inc., Foster City, CA, USA)	Significant increase in indole-3-acetic acid levels in PD
27. [215]	2021	PD	Ceramide, triacylglycerol, glycosphingolipid, fatty acyl metabolites	LC	Synapt G2-Si Q-TOF (Waters, Milford, MA, USA)	Alteration in sphingolipid metabolism, arachidonic acid metabolism, and fatty acid biosynthesis
28. [216]	2025	ALS	Phosphatidylinositol, lysosphingomyelin, phosphatidylcholine, diacylglycerol	LC	Q-TOF 6520 (Agilent Technologies, Santa Clara, CA, USA)	Identification of several key metabolites and fatty acids that can be considered prognostic markers for ALS
29. [222]	2025	ALS	Phospholipids	LC	Q Exactive Orbitrap (Thermo Fisher Scientific, San Jose, CA, USA)	Impaired citrate cycle and complex lipid metabolism
30. [211]	2023	ALS	Kynurenine pathway	LC	XEVO TQ-S MS/MS (Waters, Etten-Leur, The Netherlands)	Lower anthranilic acid levels and kynurenine-to-tryptophan ratios in ALS
31. [82]	2021	ALS	maltose, glyceric acid, lactic acid, beta-alanine, phosphoric acid, glutamic acid, ethanolamine, glycine, 2,4,6-tri-tert-butylbenzenethiol	GC	Agilent 5975C, Agilent 7890A (Agilent Technologies, Santa Clara, CA, USA)	Alteration of glycine, serine, and threonine metabolism, D-glutamine and D-glutamate metabolism, alanine, aspartate, and glutamate metabolism, beta-alanine metabolism, and pyruvate metabolism
32. [212]	2025	HD	3-hydroxykynurenine, quinolinic acid, kynurenine, anthranilic acid, kynurenic acid, tryptophan	LC	Triple quadrupole SCIEX 5500/6500 (AB SCIEX, Redwood City, CA, USA)	No dysregulation of the kynurenine pathway metabolites
33. [223]	2024	HD	24(S)-hydroxycholesterol (24S-OHC), 25-OHC, 27-OHC	LC	Triple quadrupole, SCIEX 6500 QTRAP (AB SCIEX, Redwood City, CA, USA)	Lower 24(S)-OHC levels and 24(S)/25-OHC ratios in early HD
34. [48]	2024	HD	Bloch pathway	LC	Triple quadrupole LCMS8060 (Shimadzu, Japan)	Significant downregulation of desmosterol and 24S-OHC levels
35. [206]	2024	MS	Galactose metabolism, amino sugar, and nucleotide sugar metabolism	GC	TOF Agilent 6890 (Agilent Technologies, Santa Clara, CA, USA)	Dysregulation of methyl 11,14-eicosadienoate (S), 11,14-eicosadienoic acid, L-tyrosine, 2-hydroxypentanoic acid (S), erythrose, and margaric acid

Significant alterations of fatty acyls, glycerophospholipids, and phosphosphingolipids were observed in patients with PD [31]. Elevated levels of plasma phosphatidylcholine were reported to increase the risk of PD significantly [224]. Dysregulated lipids, including sphingolipids and phospholipids, were observed in patients with dementia with Lewy bodies [225]. Diacylglycerol was significantly upregulated in the pre-symptomatic stage of AD, making it a potential biomarker for early diagnosis [32]. Significant alterations in cholesterol, including very low-density lipoproteins (VLDL) and low-density lipoproteins (LDL), such as VLDL5, LDL3, LDL4, and LDL5, were observed in both presymptomatic and symptomatic HD patients compared with controls [47]. Dysregulation of 24(S)-hydroxycholesterol (24S-OHC), 25-OHC, and 27-OHC was also observed in HD [223]. The Bloch pathway was reported to be affected by HD, leading to the downregulation of desmosterol and 24S-OHC [48]. Among carbohydrate metabolites, different studies reported dysregulation of D-galactose, D-arabitol in AD, maltose in ALS, and galactose in MS [82,206,208].

## 5. Challenges and Future Direction

### 5.1. Challenges and Limitations

MS-based metabolomics has been a powerful tool in deciphering the complex biological processes and advancing our understanding of neurodegenerative diseases. However, several challenges still limit the clinical integration of MS-based metabolomics for neurodegenerative diseases.

Data quality, standardization, and reproducibility have historically been neglected despite their important influence on MS-based metabolomics [226]. The lack of standardized methods for processing diverse biological samples, including biofluids and tissues, is a major challenge. Because of differences in initial sample processing across sample types, there is a high likelihood of variation at the sample preparation step. When it comes to neurodegenerative research, it is even more challenging because distinguishing disease-related metabolic changes from natural physiological variation is more difficult [166].

Another challenge is choosing between different analytical strategies, such as targeted and untargeted metabolomic approaches [166]. While untargeted metabolomics offers a broad view of the metabolome, it often lacks the sensitivity needed to detect low-concentration compounds and faces difficulties in confidently identifying metabolites. In contrast, targeted methods deliver precise quantification but are limited to preselected metabolites, potentially missing out on novel markers. The transition from relative abundance to absolute quantification introduces additional complexity, requiring internal standards or chemical derivatization, which can introduce analytical errors [17,19].

Beyond the laboratory bench, data processing and interpretation have been significant challenges in MS-based metabolomics for studying neurodegenerative diseases. MS platforms generate vast amounts of information, and accurately and efficiently processing raw data into biologically meaningful results requires highly advanced bioinformatics tools [227]. Managing peak detection, normalization, and statistical modeling requires a combination of expertise in chemistry, data science, and biostatistics. Even with advanced tools, no single platform can fully capture the metabolome’s vast chemical diversity [227]. As a result, many promising candidate biomarkers do not achieve clinical utility due to inadequate pre-analytical controls or the high costs and time required for large-scale prospective validation to prove their medical value.

### 5.2. Future Direction

Metabolomics is a cornerstone of systems biology, providing functional biochemical information that complements and integrates data from other omics studies. Over the last decade, advancements in MS have established it as one of the most powerful analytical platforms for both qualitative and quantitative metabolite analysis due to its high sensitivity, selectivity, and versatility, as well as its compatibility with various separation techniques.

Moving MS-based metabolomics toward clinical use for neurodegenerative disorders requires several key research initiatives. Combining metabolomics with other omics fields, such as genomics, proteomics, and transcriptomics, is a top priority. This multi-omics approach provides a more comprehensive view of disease mechanisms, enabling researchers to connect metabolic changes to specific genetic or molecular factors [228,229]. By establishing these links, we can significantly improve the biological validity of identified biomarkers.

Addressing the computational challenges of big data is equally crucial in this field. Advanced bioinformatics tools and machine learning algorithms are necessary to enhance the efficiency of data collection, feature extraction, and metabolite identification. Machine learning has shown the ability to automate complex data processing tasks while improving the accuracy of disease classification [230]. Continued research on these digital frameworks is essential for handling the growing complexity of large-scale datasets.

Furthermore, future research is needed to validate biomarkers in large-scale clinical cohorts to demonstrate their clinical correlations. Developing standardized protocols for sample collection and storage to prevent pre-analytical inconsistencies is another critical aspect. Improvements in methodology, such as more sensitive sample preparation and broader metabolome coverage, need to be taken into account. Finally, multi-omics integration should be applied at the translational level to go beyond single-metabolite markers. Creating comprehensive metabolite panels by integrating metabolomics data with neuroimaging, other clinical parameters, and molecular indicators will be more effective for disease diagnosis. Establishing proper guidelines for developing and validating these biomarkers in laboratory settings will be another crucial step in moving these discoveries from research into routine medical practice.

## Figures and Tables

**Table 1:** Summary of extraction methods, separation, ionization, and dissociation techniques for metabolites.

Workflow Phase	Technique	Description	Key Benefits	Drawbacks
Metabolite Extraction	Organic Solvent Precipitation	Using MeOH or ACN to precipitate proteins and extract small molecules	Fast and compatible with LC-MS	May miss highly volatile or polar compounds
	Liquid–Liquid Extraction	Separating analytes according to their solubility in different liquid layers	Effective cleanup; excellent for lipids	Time-consuming; results may vary
	Solid-Phase Extraction	Using a solid sorbent to trap and then wash out metabolites	High purity and selectivity	Requires specific protocol optimization
	Biphasic (Folch/Bligh-Dyer)	Two-layer extraction targeting both fats and water-soluble components	Ideal for lipidomics studies	High solvent consumption; slow process
	Cryo-Homogenization	Breaking down tissue at freezing temperatures	Prevents the breakdown of unstable metabolites	Requires specialized hardware
Analyte Separation	RPLC	Sorting compounds by hydrophobicity	Highly reliable with versatile coverage	Fails to retain highly polar molecules
	HILIC	Specifically designed for water-soluble, hydrophilic analytes	Superior for capturing polar compounds	Susceptible to interference from the matrix
	GC	Sorting volatile or chemically modified compounds	High precision and resolution	Chemical derivatization is essential
	CE	Sorting by ionic charge and molecular size	Ideal for charged species	Generally, less stable than LC
	IM	Sorting in the gas phase based on molecular size, shape, and charge	Can identify different isomers	Increases data complexity
Ionization Source	ESI	Gently converting liquid samples into gas-phase ions	Ideal for polar compounds	Prone to ion suppression
	APCI	Using gas-phase reactions to ionize molecules	Works well for moderate polar species	Less sensitive to highly polar analytes
	APPI	Using light (photons) to initiate ionization	Good for ionizing nonpolar compounds	Less efficient for strongly ionic, highly polar, and zwitterionic analytes
	MALDI	Laser-triggered ionization of a solid surface	Crucial for spatial tissue imaging	Low sensitivity; requires a large sample size
Fragmentation Techniques	CID/HCD	Breaking molecules apart through collisions or high-energy beams	Standard for structural identification	Not sufficient to reveal all structural details of the complex metabolites
	EAD/ETD	Using radicals to trigger specific fragment patterns	Offers detailed structural insights	Not available on all instruments

Abbreviations: RPLC—reverse phase liquid chromatography, HILIC—hydrophilic interaction liquid chromatography, GC—gas chromatography, CE—capillary electrophoresis, IM—ion mobility, ESI—electrospray ionization, APCI—atmospheric pressure chemical ionization, APPI—atmospheric pressure photoionization, MALDI—matrix-assisted laser desorption ionization, CID—collision-induced dissociation, HCD—higher-energy collisional dissociation, EAD—electron-activated dissociation, ETD—electron-transfer dissociation.

**Table 2:** Metabolomics data processing and analysis software.

Program	Features			Website, accessed on 18 March 2026
	Statistics	Pathway Analysis	Data Visualization	
MetaboAnalyst 6.0 [189]	Y *	Y	Y	https://www.metaboanalyst.ca/
MS-DIAL 5 [190]	Y	-	Y	https://github.com/systemsomicslab/MsdialWorkbench
MZmine 3 [191]	Y	-	Y	https://www.mzmine.org/
MassCube 1.1.10 [192]	Y	-	Y	https://github.com/huaxuyu/masscube
TraceMetrix [193]	Y	Y	Y	https://www.biosino.org/tracemetrix
SMART 2.0 [194]	Y	-	Y	https://github.com/YuJenL/SMART
Galaxy 25.0 [195]	Y		Y	https://workflow4metabolomics.usegalaxy.fr
DNEA 2023 [196]	Y	Y	Y	http://www.github.com/Karnovsky-Lab/DNEA/
WebGestalt 2024 [197]	Y	Y	Y	https://www.webgestalt.org
XCMS-METLIN 3.7.1 [198]	Y	Y	Y	https://xcmsonline.scripps.edu/
OpenMS 2026 [199]	Y	-	Y	https://www.openms.org/
MetDNA3 [200]	-	Y	Y	http://metdna.zhulab.cn/
GNPS2 [201]	-	-	Y	https://gnps2.org/
Sirius 4 [202]	-	-	Y	https://bio.informatik.uni-jena.de/sirius/

(* Y = Yes).

## Data Availability

No new data were created or analyzed in this study. Data sharing is not applicable to this article.
